# Supplementary material for: Total replacement of fishmeal with poultry by-product meal affected the growth, muscle quality, histological structure, antioxidant capacity and immune response of juvenile barramundi, Lates calcarifer
Source: PLoS One. 2020 Nov 12;15(11):e0242079. doi: 10.1371/journal.pone.0242079 (PMC7661056; doi:10.1371/journal.pone.0242079)
Supplement: Attachment — Submitted filename: PONE-D-20-07405_reviewer (1).pdf [file pone.0242079.s001.pdf]

PLOS ONE

Total replacement of fishmeal with poultry by-product meal affected the growth, muscle quality, histological structure, antioxidant capacity and immune response of juvenile barramundi, Lates calcarifer

--Manuscript Draft--

**Manuscript Number:**	PONE-D-20-07405
**Article Type:**	Research Article
**Full Title:**	Total replacement of fishmeal with poultry by-product meal affected the growth, muscle quality, histological structure, antioxidant capacity and immune response of juvenile barramundi, Lates calcarifer
**Short Title:**	Total replacement of fishmeal with poultry by-product meal in the diet of barramundi, Lates calcarifer
**Corresponding Author:**	Md Reaz Chaklader<br>Curtin University<br>Perth, WA AUSTRALIA
**Keywords:**	
**Abstract:**	The present study investigated the effects of total replacement of dietary fishmeal (FM) with poultry by-product meal (PBM), supplemented with methionine on muscle fatty acids composition, intestinal microvilli morphology, histological structure of liver, muscle, gill and intestine, liver enzymes, immune response and stress related gene in juvenile barramundi, Lates calcarifer in relation to growth and feed utilization. Juvenile barramundi (3.58±0.01g) were randomly distributed into 300L seawater recirculatory tank (25 fish/tank) and fed two formulated isonitrogenous and isolipidic diets for 6 weeks. The control diet had FM as sole animal protein source, whereas other test diet had only PBM as an animal protein source. Dietary PBM affected the fish performance and feed utilization. Liver, muscle, gill and intestinal histology showed no obvious alteration in control fed fish, however more lipid droplet and heapatic vacuolization in liver, necrotic myotome in muscle, hyperplasia in secondary lamellae in gill and short and broken folds in intestine was observed in PBM fed fish. Similar to intestinal histology, transmission electron microscopy (TEM) analysis revealed shorter and smaller microvilli height and diameter in PBM fed fish than control fed fish. Altered values of liver enzymes including AST and GLDH showed negative effects of PBM fed fish, which was supported by a significant upregulation of stress related genes, HSP70 and HSP90, as well as a significant decrease in serum immune parameters including lysozyme and bactericidal activity. In conclusion, a total substitution of FM protein by PBM negatively influenced the growth performance, liver health, histomorphology of different organs and immune status of juvenile barramundi.
**Order of Authors:**	Md Reaz Chaklader
	Muhammad A.B. Siddik
	Ravi Fotedar
**Additional Information:**	
**Question**	**Response**
**Financial Disclosure**<br><br>Enter a financial disclosure statement that describes the sources of funding for the work included in this submission. Review the submission guidelines for detailed requirements. View published research articles from *PLOS ONE* for specific examples.	Support of the trial was obtained from the Research Training Program (RTP) Stipend Scholarship, funded by Australian Government to Md Reaz Chaklader (No. 19061054-Curtin).

Powered by Editorial Manager® and ProduXion Manager® from Aries Systems Corporation

This statement is required for submission and **will appear in the published article** if the submission is accepted. Please make sure it is accurate.<br><br>**Unfunded studies**<br>Enter: *The author(s) received no specific funding for this work.*<br><br>**Funded studies**<br>Enter a statement with the following details:<br>• Initials of the authors who received each award<br>• Grant numbers awarded to each author<br>• The full name of each funder<br>• URL of each funder website<br>• Did the sponsors or funders play any role in the study design, data collection and analysis, decision to publish, or preparation of the manuscript?<br>• **NO** - Include this sentence at the end of your statement: *The funders had no role in study design, data collection and analysis, decision to publish, or preparation of the manuscript.*<br>• **YES** - Specify the role(s) played.<br><br>* typeset	
**Competing Interests**<br><br>Use the instructions below to enter a competing interest statement for this submission. On behalf of all authors, disclose any competing interests that could be perceived to bias this work—acknowledging all financial support and any other relevant financial or non-financial competing interests.<br><br>This statement **will appear in the published article** if the submission is accepted. Please make sure it is accurate. View published research articles from *PLOS ONE* for specific examples.	The authors declare no competing interests.

**NO authors have competing interests**<br><br>Enter: *The authors have declared that no competing interests exist.*<br><br>**Authors with competing interests**<br><br>Enter competing interest details beginning with this statement:<br><br>*I have read the journal's policy and the authors of this manuscript have the following competing interests: [insert competing interests here]*<br><br>* typeset	
**Ethics Statement**<br><br>Enter an ethics statement for this submission. This statement is required if the study involved:<br><br>• Human participants<br>• Human specimens or tissue<br>• Vertebrate animals or cephalopods<br>• Vertebrate embryos or tissues<br>• Field research<br><br>Write "N/A" if the submission does not require an ethics statement.<br><br>General guidance is provided below. Consult the submission guidelines for detailed instructions. **Make sure that all information entered here is included in the Methods section of the manuscript.**	The experiment were conducted at Curtin Aquatic Research Laboratory (CARL) in Curtin University, Australia in compliance with relevant guidelines and regulations set by Australian Code of Practice for the care and use of animals for scientific purposes. All methods involving fish were reviewed and approved by the Curtin University Animal Ethics Committee (ARE2018-37). Prior to handling fish, AQUI-S® was used as anaesthesia and an overdose of AQUI-S was used as euthanasia to minimise stress, pain and discomfort to the fish following the protocol of the Curtin Research Laboratories SOP of anaesthetizing and euthanizing of fish.

Powered by Editorial Manager® and ProduXion Manager® from Aries Systems Corporation

**Format for specific study types**<br><br>**Human Subject Research (involving human participants and/or tissue)**<br>• Give the name of the institutional review board or ethics committee that approved the study<br>• Include the approval number and/or a statement indicating approval of this research<br>• Indicate the form of consent obtained (written/oral) or the reason that consent was not obtained (e.g. the data were analyzed anonymously)<br><br>**Animal Research (involving vertebrate animals, embryos or tissues)**<br>• Provide the name of the Institutional Animal Care and Use Committee (IACUC) or other relevant ethics board that reviewed the study protocol, and indicate whether they approved this research or granted a formal waiver of ethical approval<br>• Include an approval number if one was obtained<br>• If the study involved *non-human primates*, add *additional details* about animal welfare and steps taken to ameliorate suffering<br>• If anesthesia, euthanasia, or any kind of animal sacrifice is part of the study, include briefly which substances and/or methods were applied<br><br>**Field Research**<br><br>Include the following details if this study involves the collection of plant, animal, or other materials from a natural setting:<br>• Field permit number<br>• Name of the institution or relevant body that granted permission	
**Data Availability**<br><br>Authors are required to make all data underlying the findings described fully available, without restriction, and from the time of publication. PLOS allows rare exceptions to address legal and ethical concerns. See the PLOS Data Policy and FAQ for detailed information.	Yes - all data are fully available without restriction

A Data Availability Statement describing where the data can be found is required at submission. Your answers to this question constitute the Data Availability Statement and **will be published in the article**, if accepted.<br><br>**Important:** Stating 'data available on request from the author' is not sufficient. If your data are only available upon request, select 'No' for the first question and explain your exceptional situation in the text box.<br><br>Do the authors confirm that all data underlying the findings described in their manuscript are fully available without restriction?	
**Describe where the data may be found in full sentences. If you are copying our sample text, replace any instances of XXX with the appropriate details.**<br><br>• If the data are **held or will be held in a public repository**, include URLs, accession numbers or DOIs. If this information will only be available after acceptance, indicate this by ticking the box below. For example: *All XXX files are available from the XXX database (accession number(s) XXX, XXX.*).<br>• If the data are all contained **within the manuscript and/or Supporting Information files**, enter the following: *All relevant data are within the manuscript and its Supporting Information files.*<br>• If neither of these applies but you are able to provide **details of access elsewhere**, with or without limitations, please do so. For example:<br><br>*Data cannot be shared publicly because of [XXX]. Data are available from the XXX Institutional Data Access / Ethics Committee (contact via XXX) for researchers who meet the criteria for access to confidential data.*<br><br>*The data underlying the results presented in the study are available from (include the name of the third party*	All relevant data are within the manuscript and its Supporting Information files.

and contact information or URL).<br>• This text is appropriate if the data are owned by a third party and authors do not have permission to share the data.<br><br>* typeset	
Additional data availability information:	

Article file Click here to download Manuscript Manuscript .docx

Total replacement of fishmeal with poultry by-product meal affected the growth, muscle quality, histological structure, antioxidant capacity and immune response of juvenile barramundi, *Lates calcarifer*

Authors: Md Reaz Chaklader[1*], Muhammad A.B. Siddik[2], Ravi Fotedar[1]

Affiliations:

[1]School of Molecular and Life Sciences, Curtin University, 1 Turner Avenue, Bently, WA, 6102, Australia

[2]Department of Fisheries Biology & Genetics, Faculty of Fisheries, Patuakhali Science and Technology University, Patuakhali-8602

*****Corresponding author

Email: mrreaz.chaklader@postgrad.curtin.edu.au

Abstract

The present study investigated the effects of total replacement of dietary fishmeal (FM) with poultry by-product meal (PBM), supplemented with methionine on muscle fatty acids composition, intestinal microvilli morphology, histological structure of liver, muscle, gill and intestine, liver enzymes, immune response and stress related gene in juvenile barramundi, *Lates calcarifer* in relation to growth and feed utilization. Juvenile barramundi (3.58±0.01g) were randomly distributed into 300L seawater recirculatory tank (25 fish/tank) and fed two formulated isonitrogenous and isolipidic diets for 6 weeks. The control diet had FM as sole animal protein source, whereas other test diet had only PBM as an animal protein source. Dietary PBM affected the fish performance and feed utilization. Liver, muscle, gill and intestinal histology showed no obvious alteration in control fed fish, however more lipid droplet and heapatic vacuolization in liver, necrotic myotome in muscle, hyperplasia in secondary lamellae in gill and short and broken folds in intestine was observed in PBM fed fish. Similar to intestinal histology, transmission electron microscopy (TEM) analysis revealed shorter and smaller microvilli height and diameter in PBM fed fish than control fed fish. Altered values of liver enzymes including AST and GLDH showed negative effects of PBM

fed fish, which was supported by a significant upregulation of stress related genes, HSP70 and HSP90, as well as a significant decrease in serum immune parameters including lysozyme and bactericidal activity. In conclusion, a total substitution of FM protein by PBM negatively influenced the growth performance, liver health, histomorphology of different organs and immune status of juvenile barramundi.

Introduction

One of the major bottlenecks for carnivorous aquafeed production is the reduced supply of global fishmeal (FM) and increased prices. Therefore, efforts have been dedicated over the last few years to investigate the use of alternative dietary protein from animal sources such as meat and bone meal [1], poultry by-product meal [2] and blood meal [3] to replace FM. Poultry by-product meal (PBM), an economical and easily available ingredients compared to FM contains higher level of protein and most of the indispensable amino acids with the exception of lysine and methionine [4, 5]. For example, Zapata, Lazo [6] resulted in a highest variation in lysine content, which decreased from 4.2 to 3.4% with increasing levels of PBM inclusion (0 to 100%) in the dry diets of juveniles *Totoaba macdonaldi*. Dietary inclusion of PBM into fish diets is also hindered by its deficiency of FA profile. Parés- Sierra, Durazo [7] reported a decreasing levels of HUFA (20:5n-3 and 22:6n-3) as well as a decreasing amounts of n-3/n-6 as PBM and poultry oil increased in the diet of rainbow trout, *Oncorhynchus mykiss*. Though a good number of studies have been successfully replaced FM with PBM for carnivorous fish production [8-10], utilization of PBM in excess of 50% affect the welfare of some marine fish species [4, 11, 12]. For example, Karapanagiotidis, Psofakis [13] reported that total replacement of FM with PBM significantly impacted the growth performance and feed utilization, concomitantly downregulating growth-regulating hormone (*ghri* and *igfi*) in the liver of gilthead seabream, *Sparus aurata*. Similarly, a decreased growth performance, protein efficiency ratio (PER) and net protein utilization (NPU) was observed in fish fed 100% of PBM [14]. Incorporation of limiting amino acids could be a good strategy to balance alternative protein amino acid profile and to modulate the growth performance of fish. Methionine, a limiting essential amino acids in PBM play an important role for protein synthesis and also essential for cysteine and taurine biosynthesis [15]. In addition, it serves as DNA methylation reaction and as a precursor of polyamines, L-carnitine, and cysteine [16, 17].

Assessing serum biochemistry, antioxidants and immune indices is important to evaluate the health status of farmed fish. In many species, a total replacement of FM protein with other ingredients in aquafeeds has negative repercussion on immune status [18], serum biochemistry and antioxidants of fish. Many earlier studies have reported the significant effect of dietary alternative protein ingredients on immune response of European seabass, *Dicentrarchus labrax* [19], turbot, *Scophthalmus maximus* L.[20], red sea bream, *Pagrus major* [21], gilthead sea bream, *Sparus aurata* L.[22], sunshine bass, *Morone chrysops* × *M. saxatilis* [23] and blood biochemistry of hybrid grouper, *Epinephelus fuscoguttatus*♀× *Epinephelus lanceolatus*♂ [18], Nile tilapia *Oreochromis niloticus* [24] and Siberian sturgeon *Acipenser baerii* [25]. Hence, it is crucial to understand how total replacement of FM with PBM may induce the fish serum biochemistry, antioxidant and immune status.

During dietary modification, it is important to consider that replacement of FM with potential ingredients do not exert adverse effects on the welfare of the tested species, as the welfare of fish in captive condition has been a growing concern over the decades [26-28]. Histological approach is one of the important frontline tools to assess the health status of fish which can be achieved by evaluating the morphological status of different organs. Though this tool has been extensively using to assess the health condition of wild fish in response to aquatic pollution [29-31], it has gained importance recently to evaluate the welfare of farmed animals along with other parameters including growth, serum biochemistry and immune response. However, histological evaluation of different organs in farmed to higher inclusion of PBM is still scarce.

Barramundi or Asian sea bass (*Lates calcarifer*) is a commercial important fish species due to its good meat quality, ability to tolerate a wide range of salinity and ability to adapt in versatile farming environment [32]. It is popularly cultivated both in freshwater and sea water in Malaysia, Thailand, Taiwan, Indonesia, Saudi Arabia and Australia, contributing USD 320 millions globally [33]. A preliminary study was conducted in our laboratory to assess growth, gut microvilli morphology, fatty acids and amino acids composition of juvenile barramundi when fed 75 and 100% PBM either bioprocessed or unprocessed [32], however, so far, a thorough growth study including serum biochemical parameters, immune response, stress related genes, antioxidant capacity, histological evaluation of different organs and intestinal mucosal morphology is still limited. Therefore, to evaluate the total replacement of FM with PBM, the present study was conducted over 42 days on juvenile barramundi and serum biochemical parameters, immune response, stress related genes, antioxidant capacity,

histological evaluation of different organs and intestinal mucosal morphology was evaluated when fed PBM as a main protein source.

Materials and methods

Animal ethical statement

The experiment were conducted at Curtin Aquatic Research Laboratory (CARL) in Curtin University, Australia in compliance with relevant guidelines and regulations set by Australian Code of Practice for the care and use of animals for scientific purposes. All methods involving fish were reviewed and approved by the Curtin University Animal Ethics Committee (ARE2018-37). Prior to handling fish, AQUI-S® was used as anaesthesia and an overdose of AQUI-S was used as euthanasia to minimise stress, pain and discomfort to the fish following the protocol of the Curtin Research Laboratories SOP of anaesthetizing and euthanizing of fish.

Experimental diets

All the ingredients required for formulating test diets were purchased from the Special Feeds, 3150 Great Eastern Hwy, Glen Forrest, WA. Two isonitrogenous and isolipidic containing approximately 47% crude protein and 12% crude lipid were prepared to meet the nutritional requirement of juvenile barramundi [34]. FM and PBM were used as main protein source and canola oil and cod liver oil was used as lipid resource. A control diet was prepared based on FM and another diet was formulated by replacing 100% of FM with PBM along with the supplementation 0.40% methionine. The test diets were formulated in compliance with the standard protocol of CARL. Briefly, all the dry ingredients were mixed homogenously using a food mixture (Hobart Food equipment, Australia) before blending with fish oil and distil warm water to make a stiff dough. The dough were passed through a mincer to make 3 mm pellets, then spread out and dried in an oven at 60°C for 36 hours. After drying, pellets were sealed in plastic bags before refrigerating at 4°C until used in the feeding trial. Fatty acids and amino acids profile of experimental diets are shown in Table 2.

Table 1. Formulation and proximate composition of test diets supplementing with different fish protein hydrolysates for juvenile barramundi over period of 8 weeks.

	Control	100PBM
Ingredients[a] (g/100g DM)		
Poultry meal[b]	0.00	69.50
Canola oil	1.00	3.00
Fish meal[c]	72.00	0.00
Corn/wheat starch	7.00	7.00
Lecithin - Soy (70%)	1.00	1.00
Vitamin C	0.05	0.05
Dicalcium Phosphate	0.05	0.05
wheat (10 CP)	16.90	11.50
Methionine	0.00	0.40
Vitamin premix	0.50	0.50
Salt (NaCl)	1.00	1.00
Cod liver oil	0.50	6.00
Proximate composition (% dry weight)		
Crude Protein	47.88	47.86
Crude Lipid	12.59	12.71

[a] Specialty Feeds, Glen Forrest Stockfeeders, 3150 Great Eastern Highway, Glen Forrest, Western Australia 6071

[b] PBM (Poultry by-product meal): 67.13% crude protein,13.52% crude lipid and 13.34% ash

[c] Fishmeal: 64.0% crude protein, 10.76% crude lipid and 19.12% ash.

Table 2. Fatty acids (% of total fatty acids) and amino acids (g/100g) composition of control and experimental diet used to feed barramundi.

Fatty acids	Control	100PBM	Amino acids	Control	100PBM
C8:0	1.39	3.53	Hydroxyproline	1.7	3.2
C10:0	0.59	3.03	Histidine	2.4	1.8
C11:0	0.00	0.00	Taurine	0.5	0.5
C12:0	2.73	7.11	Serine	5.3	5.0
C13:0	1.53	2.20	Arginine	4.5	4.9
C14:0	131.63	342.45	Glycine	13.2	16.4
C14:1n5	1.52	11.32	Aspartic acid	8.8	7.8
C15:0	41.42	34.66	Glutamic acid	11.7	12.1
C15:1	1.19	7.01	Threonine	4.9	4.2
C16:0	1161.21	2090.88	Alanine	9.4	9.1
C16:1n7	165.22	435.58	Proline	6.1	7.3
C17:0	118.84	76.62	Lysine	6.2	5.3
C17:1	31.53	38.14	Tyrosine	2.0	1.8
C18:0	448.54	560.33	Methionine	2.4	1.8
C18:1cis+trans	1158.94	3800.14	Valine	5.6	5.2
C18:2 trans	6.95	6.71	Isoleucine	4.3	3.8
C18:2 cis	624.01	1081.49	Leucine	7.6	6.9

C18:3n6	8.84	10.58	Phenylalanine	3.3	3.0
C18:3n3	120.20	285.67			
C18:4n3#	30.30	148.70			
C20:0	22.44	32.83			
C20:1	79.86	483.71			
C20:2	14.57	17.04			
C21:0	9.09	10.50			
C20:3n6	15.50	18.76			
C20:4n6	112.83	43.18			
C20:3n3	8.29	7.75			
C22:0	14.10	17.23			
C20:5n3	178.50	278.99			
C22:1n9	9.44	44.58			
C22:2	1.05	2.38			
C23:0	27.89	34.91			
C22:4n6#	91.14	16.39			
C24:0	0.00	0.00			
C22:5n3#	63.30	64.60			
C24:1	34.51	53.10			
C22:6n3	908.53	455.23			
∑SFA	1981.40	3216.29			
∑MUFA	1480.69	4862.27			
∑PUFA	2184.01	2437.48			
∑n-3	1309.12	1240.95			
∑n-6	228.31	88.91			
∑n-3/n-6	5.73	13.96			

Eicosapentaenoic acid, EPA; DHA, docosahexaenoic acid, sum of saturated fatty acids, ∑SFA; sum of monounsaturated fatty acids, ∑MUFA; sum of polyunsaturated fatty acids, ∑PUFA; sum of omega-3 polyunsaturated fatty acids, ∑n-3 PUFA; sum of omega-6 polyunsaturated fatty acids, ∑n-6 PUFA.

Fish husbandry and management

200 juvenile barramundi were obtained from Australian Centre for Applied Aquaculture Research, Fremantle (ACAAR), Australia in oxygenated plastic bag. Prior to commencing trial, all fish were stocked into two fibreglass tanks (300 L) filled with ocean water and fed a commercial diet (470 g protein kg^{-1} diet and 20.0 MJ kg^{-1}dietary gross energy) twice daily for two weeks to adapt them to CARL experimental facilities and conditions. Following acclimation, 180 healthy fish averaging (3.58±0.01g) were randomly distributed into six 300-L tanks, containing 250 L water in each tank. Therefore stocking number of barramundi juveniles in each tank were 25. Each tank were equipped with an aerator, electric heater and external bio-filter (Astro® 2212, China) to maintain DO, temperature and other water quality

parameters at optimal level as reported by Siddik, Howieson [35]. Hence, the temperature was maintained at 27.90–29.20 °C, dissolve oxygen (DO) at 5.92–7.42 mgL^{-1}, salinity at 32–36 ppt and photoperiod as 14:10 h LD. Commercial test kits were used to test Ammonia nitrogen (<0.50 mgL^{-1}) and nitrite (<0.50 mgL^{-1}) level regularly. Each test diet had three replicates and fed by hand twice daily at 8.00 am and 6.00 pm to visual satiety levels for 42 days. Uneaten feed, if any, was collected by siphoning to calculate feed intake and number of dead fish were monitored daily to assess the fish survival rate. After 42 days, all fish were starved for 24 h prior to weighing individually to analyse the growth performance.

Fatty acids profile

Fish muscle (one fish/tank, three fish/dietary treatment) were used for fatty acids analysis. Fish muscle were filleted, wrapped with aluminium foil and freeze dried. The fatty acids profile of experimental diets and fish flesh was carried out following the protocol of O'Fallon, Busboom [36] and Siddik, Chungu [32].

Histological and transmission electron micrograph (TEM) analysis

After 42 days of feeding, one fish from each tank was randomly euthanized with AQUI-S at 175 mg/L and sacrificed to excise liver, muscle, gill and intestine for histological and TEM evaluation in response to test diets. For histological analysis, sample of all organs were fixed immediately in 10% buffered formalin, subsequently dehydrated with series of ethanol, infiltrated in xylene and embedded in paraffin wax, as per standard histological protocols. Section of approximately 5 µm thickness were stained with Periodic Acid-Schiff (PAS) and digitally photographed under a light microscope (BX40F4, Olympus, Tokyo, Japan).

For TEM analysis, freshly collected intestinal samples washed in 2.5% glutaraldehyde buffered in 1x PBS at pH 7.4 before performing secondary fixation in 1% OsO4 (80 W 2 min on, 2 min off, 2 min on), dehydrating in ethanol (50, 70, 95 and 100% at 250 W, 40 seach) and infiltrating finally with epoxy resin in acetone (Procure 812, Proscitech) (1:3, 1:1, 3:1ratios at 250 W, 3 min each). Samples were processed as described in the earlier study in our lab [2] and screened a LaB6 TEM (JEOL2100, Japan) at 120 kV. The electron micrographs obtained from TEM analysis at 30,000 magnification were analysed using ImageJ (National Institute of Health, USA) to determine microvilli length and diameter.

Antioxidant status assessment

The enzyme activities of serum malondialdehyde (MDA), catalase (CAT) and glutathione peroxidase (GPx) were measured with commercial assay kits following the manufacturer's instructions.

Serum biochemistry and immunity

Fish were captured gently at 42 days post-feeding, immediately dipped in a bucket containing 8 mg l^{-1} of AQUI-S® and blood were taken by puncturing caudal vessel using 1 mL non-heparinized syringes (22G). Blood were allowed to clot for 24h at room temperature, centrifuged for 15 min at 3000 rpm and 4 °C, the serum collected and stored immediately at -80°C for the analysis of serum biochemical parameters and immune parameters. Serum clinical chemistry and immune related parameters were analysed according to the protocol of our earlier study [37].

RNA extraction and qRT-PCR analysis

Liver from control and PBM fed fish were aseptically collected after euthanizing (AQUI-S, 175 mg l^{-1}) the fish and preserved in RNA Later (Sigma-Aldrich, Germany) at - 80°C until RNA extraction. Five milligram of liver tissue stored in RNA Later was used for RNA extraction using RNeasy Mini Kit (Qiagen, Hilden, Germany) according to manufacturer protocol. The quality of RNA was checked by gel electrophoresis and, the purity and quantity was determined gel electrophoresis before synthesizing complementary DNA (cDNA) from 1 µg of total RNA using Omnicript RT kit (Qiagen, Hilden, Germany) following the instruction of manufacturer's company. qRT-PCR on stress related genes were performed by PowerUp™ Cyber Green Master Mix (Thermo Scientific, USA) with 7500 Real-Time PCR System (Applied Biosystems, USA) and data were normalised against housekeeping genes, *18S rRNA* and *Ef1-a*, (Table 3) and calculated using $2^{-\Delta\Delta ct}$ method.

Table 3. Primers of qPCR used in the experiment

Genes	Sequences (5′ - 3′)	Product size	Tm (◦C)	
Heat shock protein kDa70, HSP70	F: AAGGCAGAGGATGATGTC<br>R: TGCAGTCTGGTTCTTGTC	186	59	Mohd-Shaharuddin, Mohd-Adnan [38]
Heat shock protein kDa90, HSP90	F: ACCTCCCTCACAGAATACC<br>R: CTCTTGCCATCAAACTCC	197	59	Mohd-Shaharuddin, Mohd-Adnan [38]
18S rRNA, 18S	F:TGGTTAATTCCGATAACGAACGA<br>R: CGCCACTTGTCCCTCTAAGAA	94	59/60	Mohd-Shaharuddin, Mohd-Adnan [38]
Elongation factor-1α, ef1α	F: AAATTGGCGGTATTGGAAC<br>R:GGGAGCAAAGGTGACGAC	83	59/60	Mohd-Shaharuddin, Mohd-Adnan [38]

Calculation and statistics

Specific growth rate (SGR), feed conversion ratio (FCR) and total feed intake (TFI) was calculated using the following equations-

$$\text{Specific growth rate (SGR, \% / d)} = [(\ln(\text{final body weight}) - \ln(\text{pooled initial weight}))/\text{Days}] \times 100$$

$$\text{Total feed intake (TFI, g/fish)} = [(\text{dry diet consumd})/(\text{number of fish})]$$

$$\text{Feed conversion ratio (FCR)} = [(\text{dry feed fed})/(\text{wet weigth gain})]$$

$$AI = (aC12{:}0 + bC14{:}0 + C16{:}0)/(dp + eM + FM')$$

$$TI = (C14{:}0 + C16{:}0 + C18{:}0)/[(nM + nM' + p(n6) + q\,(n3) + (n3/6)]$$

All data were represented as mean±SE. The differences between control and PBM fed fish in all data were determined by unpaired student *t*-test at the significance level of 0.05 < *P* < 0.001. Percent survival at the termination of feeding trial was plotted using Kaplan-Meier survival method with Log-rank (Mantel-Cox) test.

Results

Growth performance, feed utilization and survival

Fish growth, feed intake and survival rate in response to 42 days feeding trial are presented in Figure 1. In contrast to control, PBM diet negatively influenced the FBG (Figure 1A) (t = 16.11, df = 127, P < 0.0001) and SGR (Figure 1B) (t = 16.14, df = 127, P < 0.0001) which was supported by increased FCR (Figure 1C) (t = 2.841, df = 4, P = 0.047) and lower feed intake (Figure 1D) (t = 2.981, df = 4, P = 0.041). Survival plot with 95% confidence (Figure 1E), as drawn by Kaplan-Meier survival analysis at the end of the 42 days trial decreased significantly in PBM fed fish than the control (χ^2_{100PBM} = 4.514, df = 1, P = 0.034).

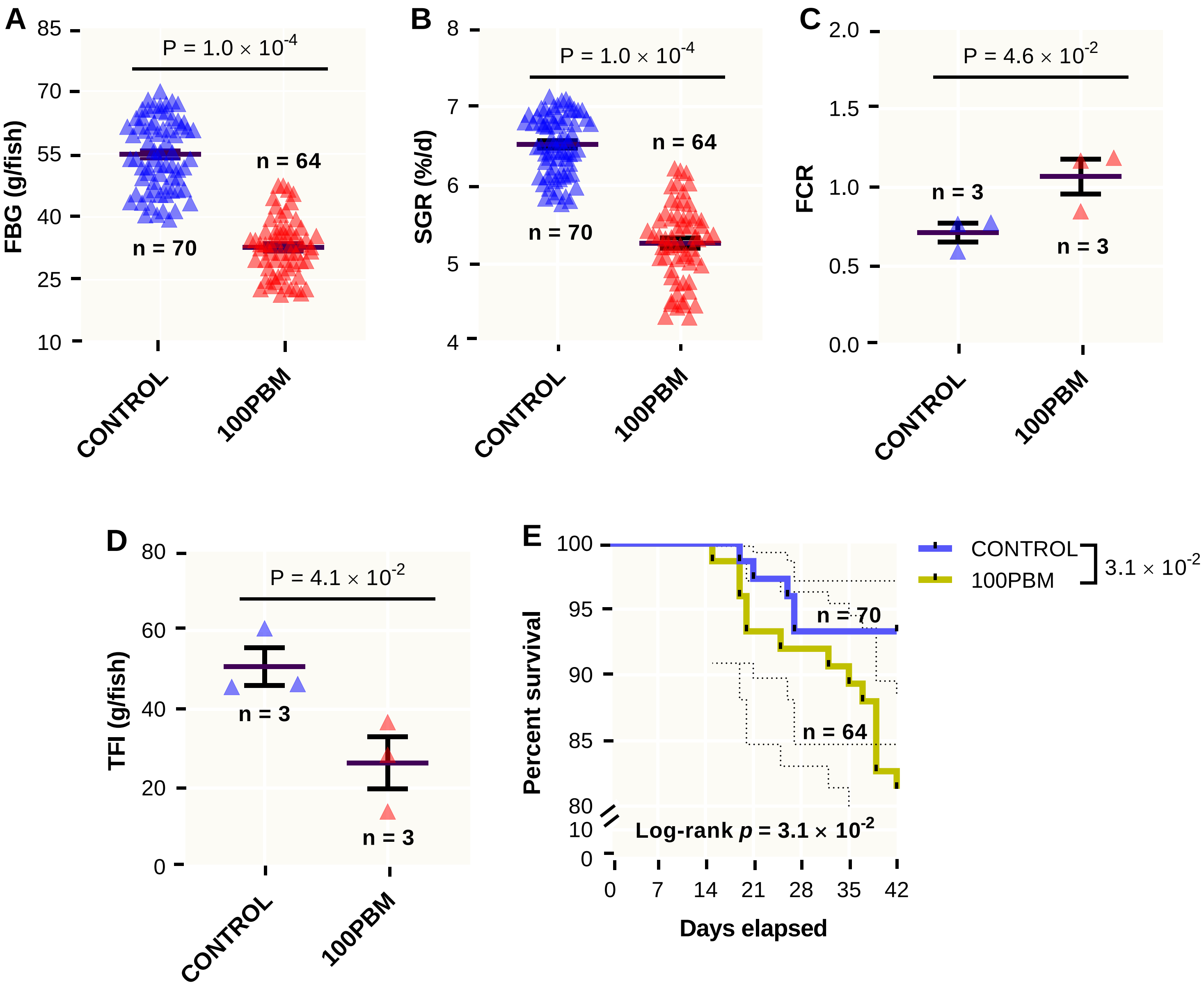

Fig 1. Fish performance including (**A)** final body weight (FBW) and (**B)** specific growth rate (SGR), (**C**) feed conversion ratio (FCR), (D) total feed intake (TFI), and (E) survival rate based on Kaplan-Meier survival analysis with Log-rank (Mantel-Cox) test of juvenile barramundi after 42 days feeding with either basal diet or total replacement of PBM. Dotted line in survival plot indicates 95% confidence interval. *P* values indicate significant at 0.05, 0.01 and 0.001, followed by an unpaired student t-test.

Muscle fatty acids composition

The FAs profile of barramundi muscle at the termination of 42 days trial were clearly influenced by 100PBM (Table 3). Dietary inclusion of 100PBM significantly augmented total SFA and ∑n-6, but worsened total MUFA, DHA and ∑n-3/∑n-6 ratio. Considering lipid indexes, PBM diet significantly increased AI with no significant effect on TI.

Table 3 Fatty acids of barramundi muscle when fed control and test diet over a period of 42 days.

	Control	100PBM	P-value
C10:0	0.78±0.03	4.31±0.06***	0.00
C12:0	1.02±0.09	291.96±1.27***	0.00
C13:0	0.60±0.00	0.75±0.09***	0.00
C14:0	67.46±0.77	213.36±0.48***	0.00
C14:1n5	0.97±0.03	6.30±0.06	0.21
C15:0	17.76±0.63	17.81±0.21	0.27
C15:1	0.00±0.00	0.18±0.20***	0.00
C16:0	713.65±10.19	1253.24±68.93***	0.00
C16:1n7	130.78±1.90	286.88±2.21***	0.00
C17:0	44.02±0.52	40.29±0.31***	0.00
C17:1	22.70±0.31	22.98±1.46	0.96
C18:0	278.21±4.07	442.18±5.83	0.96
C18:1cis+trans	859.76±5.73	2961.30±69.99	0.37
C18:2 trans	17.50±15.25	6.72±.38	0.42
C18:2 cis	320.82±3.44	1229.36±17.46**	0.00
C18:3n6	17.51±1.42	51.44±6.52*	0.01
C18:3n3	55.12±0.38	213.57±3.10***	0.00
C18:4n3	17.01±1.06	38.00±7.77***	0.00
C20:0	8.95±0.22	16.93±1.82**	0.00
C20:1	36.62±0.38	111.68±2.97*	0.01
C20:2	8.84±0.03	15.35±1.41	0.85
C21:0	4.05±0.18	6.12±0.56	0.86
C20:3n6	25.68±0.57	49.16±2.84	0.00
C20:4n6	92.29±1.90	114.06±3.54***	0.00
C20:3n3	4.61±0.09	6.10±0.81***	0.00

C22:0	3.58±0.03	7.33±0.49**	0.00
C20:5n3 (EPA)	109.31±1.78	113.69±1.31	0.52
C22:1n9	4.03±0.03	9.18±2.58	0.55
C22:2	0.00±0.00	1.00±0.00***	0.00
C23:0	13.39±0.68	25.84±1.87***	0.00
C22:4n6	62.67±1.07	23.06±0.26*	0.01
C22:5n3	71.50±0.95	78.82±0.44*	0.03
C24:1	11.81±0.12	14.05±0.69	0.00
C22:6n3 (DHA)	683.13±12.43	370.84±1.99***	0.00
∑SFA	1153.45±15.14	2409.95±21.74*	0.04
∑MUFA	1065.69±8.25	3427.59±67.50	0.11
∑PUFA	1142.86±19.58	1067.93±17.29*	0.01
∑n-3	940.67±15.92	839.03±11.05	0.05
∑n-6	198.15±3.87	254.72±4.82***	0.00
∑n-3/∑n-6	4.75±3.87	3.29±0.02**	0.00
AI	0.32±0.00	0.32±0.01*	0.01
TI	0.27±0.00	0.33±0.01	0.05

Poultry by-product meal, PBM; saturated fatty acids, SFA; monounsaturated fatty acids, MUFA; polyunsaturated fatty acids, PUFA; atherogenicity, AI and thrombogenicity, TI.

P values indicate significant at P < 0.05, 0.01 and 0.001, followed by an unpaired student t-test.

Histomorphology

Total replacement of FM with PBM dysregulated the histological structure of liver, muscle, gills and intestine **(Fig 2A-H).** The liver of control (**Fig 1A**) fed fish showed higher pigmentation of hepatocyte cytoplasm, indicating higher amount of glycogen, while the liver of PBM fed fish (**Fig 1B**) fed fish showed less hepatocyte cytoplasm pigmentation, indicating less amount of glycogen with more lipid vacuolization. Healthy and normal myotome were observed in the muscle of fish fed control diet (**Fig 1C**) but necrotic myotome was found in fish fed PBM diet (**Fig 1D)**.

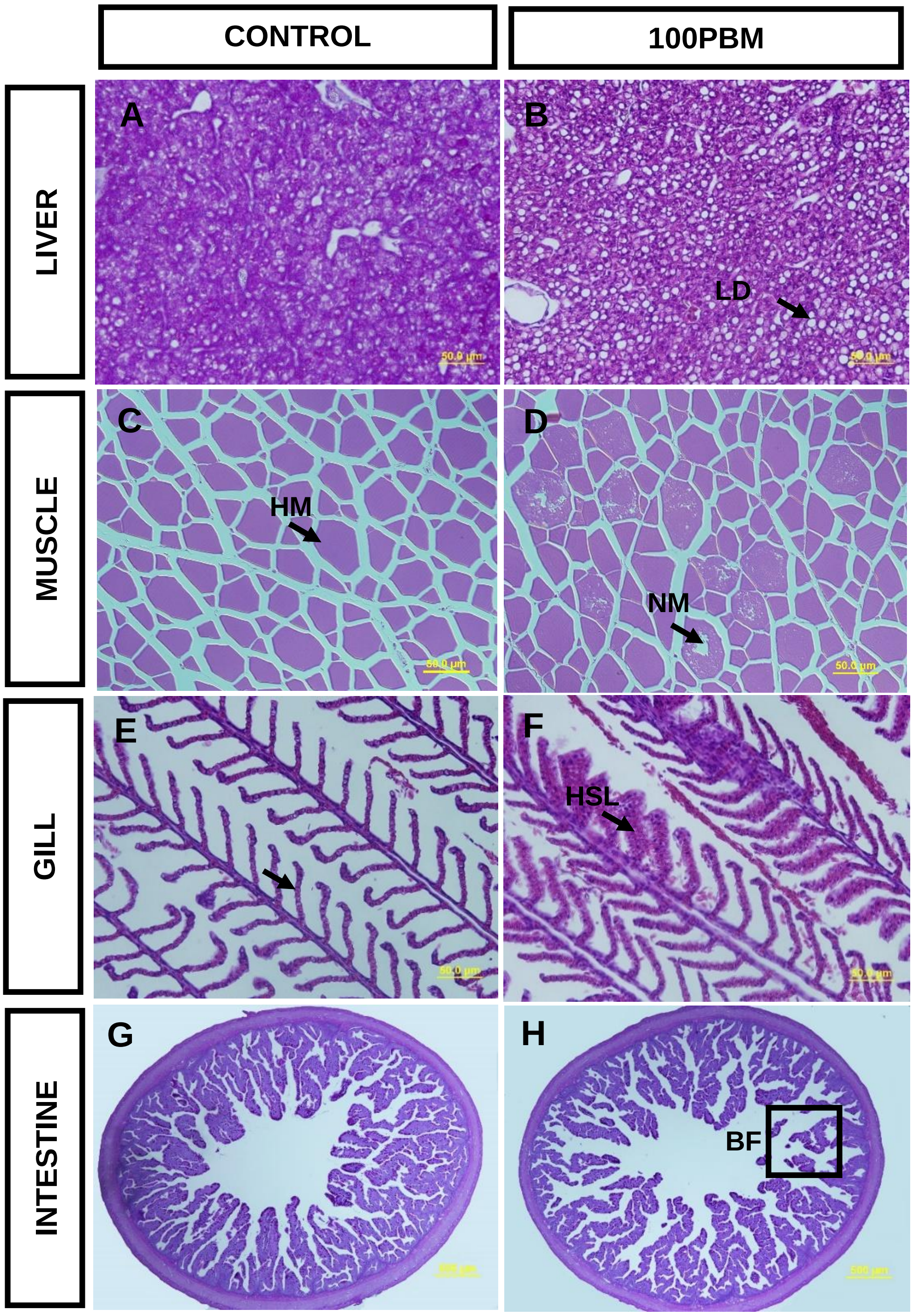

Fig 2. Liver, muscle, gill (PAS stain; 40 × magnification; scale bar = 50 µm) and distal intestine (PAS stain; 4 × magnification; scale bar = 500 µm) sections of juvenile barramundi fed control and 100PBM at the end of 42 days of feeding trial.

Intestinal morphology

The distal intestine of barramundi fed control (Fig 3A) and PBM (Fig 3B) was examined by transmission electron microscope. Microvilli height (Fig 3D) (t = 6.727, df = 28, $P < 0.0001$) and diameter (Fig 3E) (t = 3.494, df = 28, $P = 0.0016$) of barramundi fed PBM was significantly lower than barramundi fed control diet.

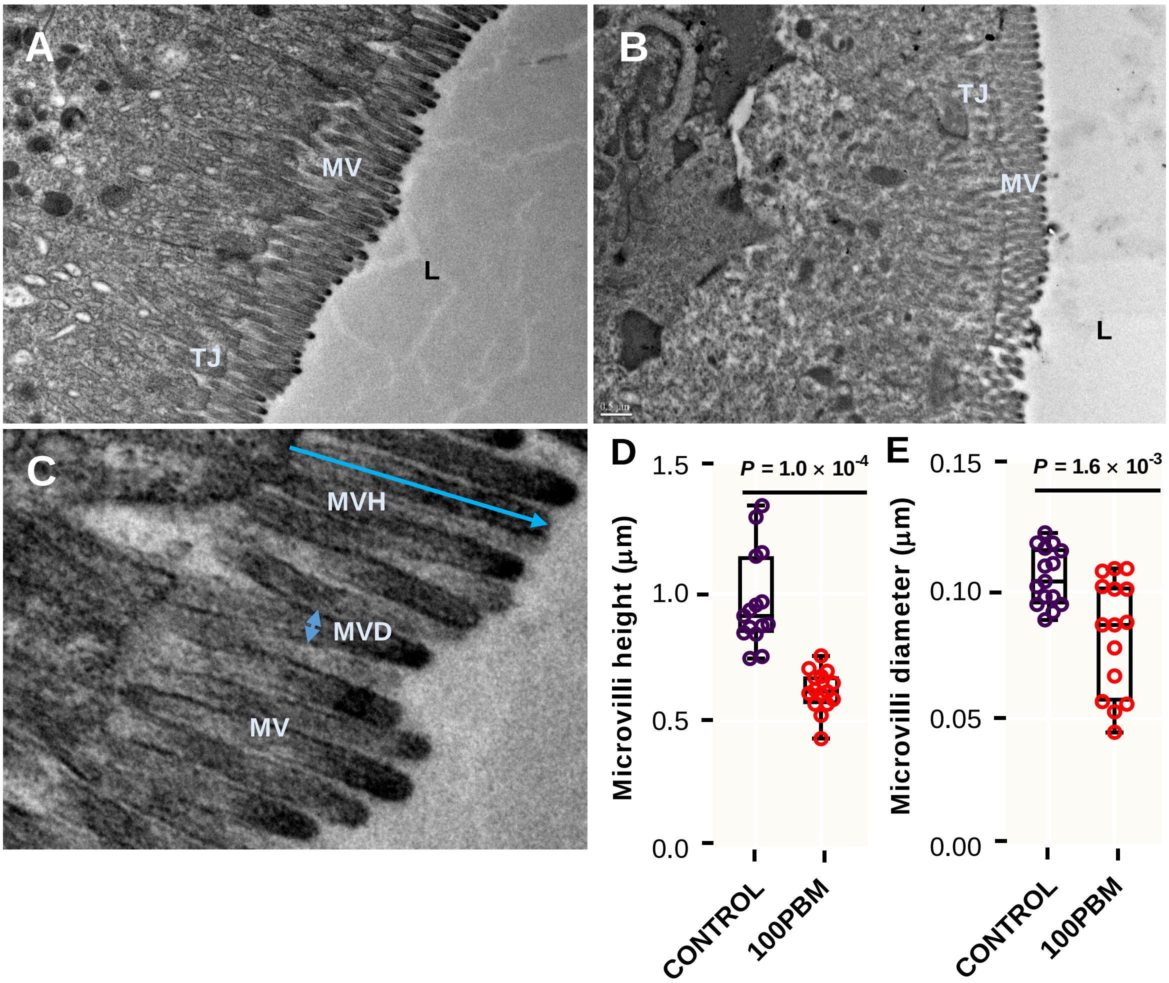

Figure 3 observation of TEM in the intestine of juvenile barramundi fed Control (A) and PBM (B) at the end of 42 days of feeding trial. (C) microvilli height and diameter measurement and comparison of microvilli height and diameter (panel D & E), performed by an unpaired student t-test at P <0.05 and 0.01.

Liver enzymes, immunity and stress related genes

Liver enzymes (AST and GLDH), immune response including serum lysozyme and bactericidal activity and stress related genes were significantly induced by experimental diets **(Fig 4).** AST and GLDH in 100PBM was significantly higher that control (t = 2.268, df = 10, P = 0.047 and t = 3.199, df = 10, P = 0.010) (Fig 1A and 1B), while serum lysozyme decreased significantly in 100PBM compared to control (t = 2.842, df = 10, P = 0.018) (Fig 1C). Meanwhile, none of the diets had significant effects on bactericidal activity (t = 1.572, df = 10, P = 0.147) (Fig 1D). In line with liver enzymes, similar results were observed in HSP70 and HSP90 when compared with control (t = 2.905, df = 10, P = 0.016 and t = 5.102, df = 10, P = 0.001) (Fig 1E and 1F).

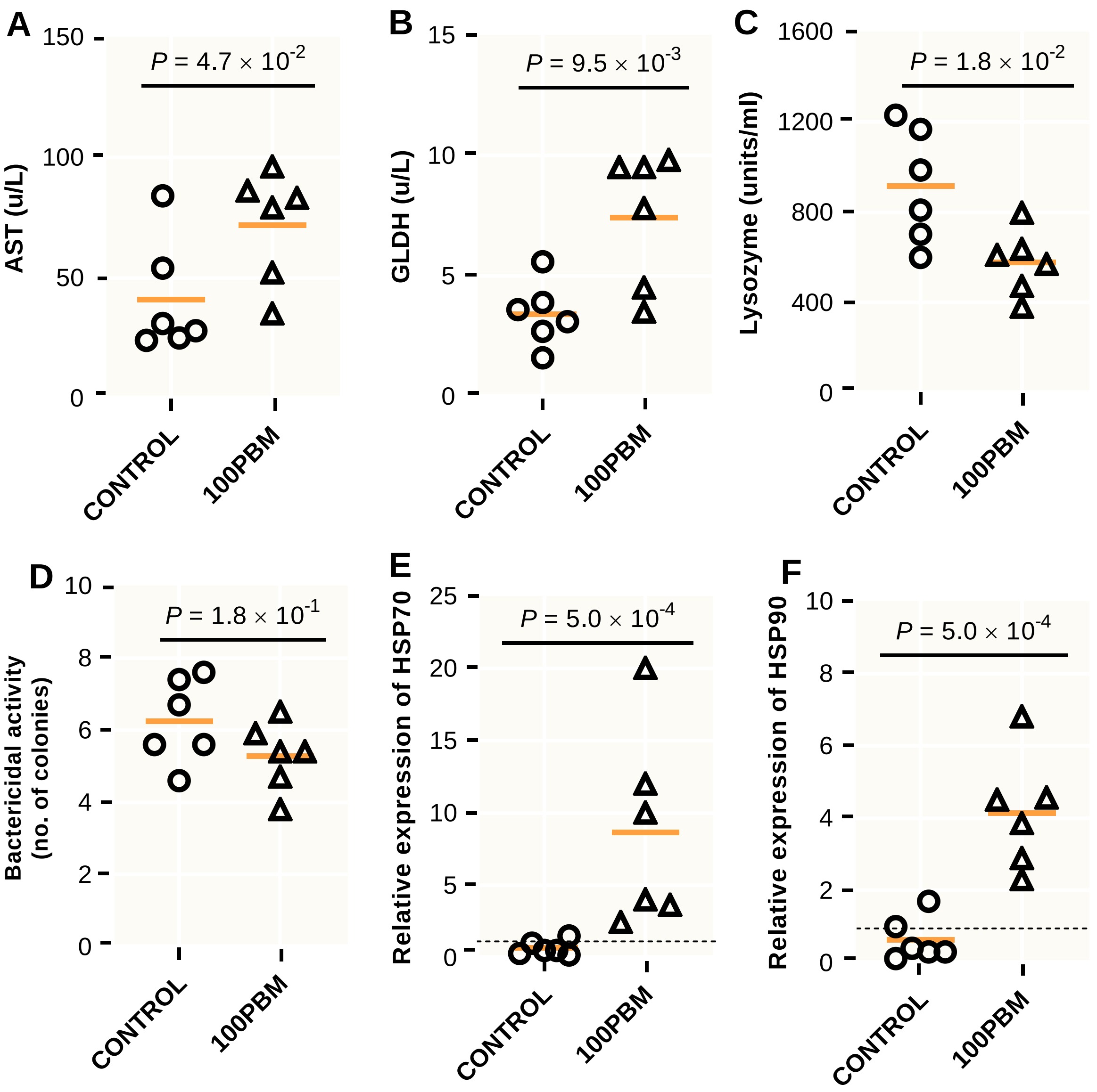

Fig 4. AST, Serum aspartate aminotransferase (A) and GLDH, glutamate dehydrogenase (B), lysozyme (C), bactericidal activity (D) and heat shock related gene including HSP70 (E) and HSP90 (F) in the liver of juvenile barramundi after 42 days feeding with either basal diet or total replacement of PBM. P values indicate significant at $P < 0.05$, 0.01 and 0.001, followed by an unpaired student t-test.

Antioxidant activity

Antioxidant activities of blood serum were significantly affected by total inclusion of PBM. Serum GPx activity declined significantly in 100PBM ($t = 2.833$, $df = 10$, $P = 0.017$) (Fig 5A), while MDA increased significant in 100PBM ($t = 2.251$, $df = 10$, $P = 0.048$) (Fig 5B) with respect to control.

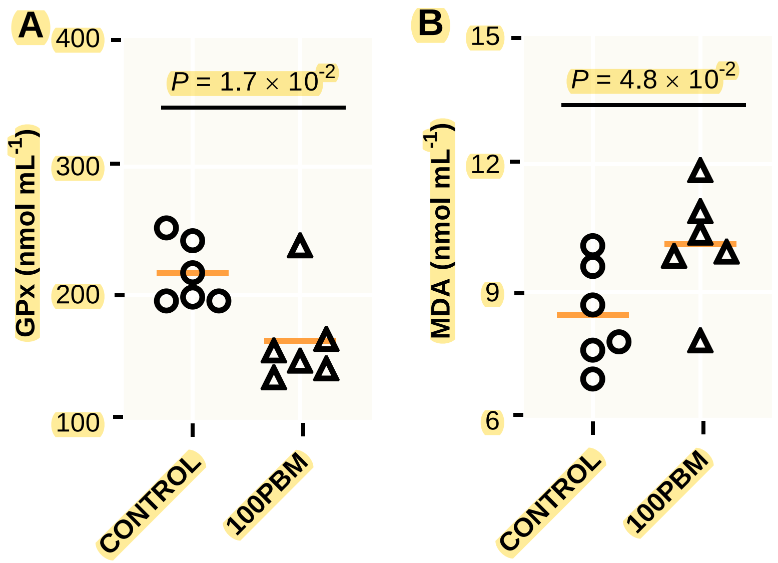

Fig 5. (A) MDA (nmol/mg protein) (B) GPx (U/mg protein) and (C) CAT (U/mg protein) in the serum of juvenile barramundi after 42 days feeding with either basal diet or total replacement of PBM. P values indicate significant at $P < 0.05$, 0.01 and 0.001, followed by an unpaired student t-test.

Discussion

A few studies have been devoted to increase the inclusion level of PBM, at the expense of FM in carnivorous fish. Though, the results are mixed as in some species, a total replacement of PBM is possible without imposing deleterious effects on the welfare of the host fish [8, 10, 39], while inclusion of PBM exceeding 50% have impacted welfare of many fish [4, 11, 13]. To understand the underlying reasons for these mixed results, the present study was carried out over 42 days to investigate why 100FM cannot be replaced with PBM in the diet of juvenile barramundi using different approaches including biometrics, light microscopy, transmission electron microscopy and qPCR. Similar to the previous study in our lab [32], growth, feed utilization, FCR and survival of juvenile barramundi was reduced when fed PBM,

supplemented with methionine. Profound deterioration in the growth performance of gibel carp, *Carassius auratus gibelio* was observed when fed 100% animal protein containing PBM and meat and bone meal (MBM), supplemented with methionine and lysine [40]. The same authors also reported that some nutritional superiority or enhanced palatability in FM that could not be met up by PBM and MBM regardless of methionine and lysine supplementation provided a biological benefit for gibel carp. Similarly, due to deficiency of AAs (methionine and lysine) and EFA (EPA and DHA), the depressed growth performance was observed in totoaba juveniles, *Totoaba macdonaldi* [6] and gilthead seabream, *Sparus aurata* [13] when fed with 100PBM. Higher inclusion of PBM protein reduce most of the essential amino acids especially lysine and methionine [4, 11] and also essential fatty acids (EFA) including n-3 LC-PUFA, EPA and DHA [41, 42] in the diet of fish, which reflected in the FAs and AAs profile of 100PBM diet. These deficiency could depress the growth performance and survival in 100PBM juvenile barramundi. However, these findings contradict with the results of Panicz, Żochowska- Kujawska [39] who reported no adverse effects of 100PBM on the growth and biometry indices of female tenches, *Tinca tinca*. This discrepancies might be due to use different fish species and culture system or nutritional composition of PBM as it varies from batch to batch or suppler companies [37].

FAs composition of diet affected the FAs composition of fish muscle or meat which have been reported in many fish species [43-45]. In addition, FA profile of fish is a crucial attribute in nutritional study as some FAs, in particular, MUFA and PUFA promote the consumer health [46]. In the present study, PBM increased the total SFAs content than the control. Higher level of SFAs are considered to be unhealthy for the cardiovascular system and, have been associated with coronary heart disease by imposing effects on cholesterol metabolism and higher LDL cholesterol levels [47]. MUFAs and PUFAs content are important for human health and its deficiency have been correlated with higher risk of cardiovascular disease by increasing inflammation, triglycerides and total cholesterol levels [47, 48] as well as neurological disease particularly myocardial infarction and stroke [49]. PUFAs particularly omega-3 and omega-6 were negatively induced by 100PBM, indicating that 100PBM fed fish may have less beneficial effect on consumer. In line with our present findings, 100PBM was lacking in essential fatty acids (EFAs) and also worsened the EFAs in the muscle of totoaba juveniles, *Totoaba macdonaldi* [6]. Similar results were also observed in the muscle of juvenile barramundi, *L. calcarifer* when fed 100PBM [32]. Lipid indexes including AI and TI estimated based on SFA, MUFA and PUFA indicate the suitability for human consumption and it value greater than 1

associated with cardiovascular disorders. 100PBM had negative effects on AI while TI showed no significant variation with values less than 1.0, still being considered healthy for human consumption [50].

AST and GLDH is an important enzymes to evaluate the liver health status of fish, leaking in blood serum significantly from the damaged cell membrane of liver when fish expose to stressful condition. In the present study, PBM diet significantly increased the levels of AST and GLDH in the serum of barramundi than the control, which are concomitant with the histopathological damage of liver tissue, thus indicated that liver health of fish was affected by total replacement of FM with PBM. Likewise, plasma ALT was negatively impacted by the inclusion of animal protein blend (APB) (20% to 80%) in diet of hybrid grouper, *Epinephelus fuscoguttatus*♀× *Epinephelus lanceolatus*♂ while AST augmented significantly in 80% APB fed fish [18]. However, Panicz, Żochowska- Kujawska [39] reported insignificant effects on blood biochemical parameters of juvenile tenches, *Tinca tinca* 86 days post-feeding with graded levels of PBM (25.7 to 100%).

To further clarify the effects of PBM on the liver function of juvenile barramundi, heat shock related genes including HSP70 and HSP90 were examined. HSP70 and HSP90 are two important stress related protein and their expression level elevate significantly when fish expose to different stress, including pathogenic infection, crowding, poor water quality and nutritional deficient diet [51-53]. In the present study, both HSP70 and HSP90 upregulated significantly in the liver of barramundi that received 100PBM compared to control, which indicate that 100% inclusion of PBM could exert stress to fish.

In fish, immune functions of immune organs are strongly associated with the presence and activity of a unique array of molecules including lysozyme, complement proteins, immunoglobulins [54-56] and bactericidal activity which are influenced by dietary modifications. Serum lysozyme and bactericidal activity were negatively triggered by 100PBM which supported the findings of Subhadra, Lochmann [57], [58] who reported aggravated levels of complement and lysozyme activity in PBM treated largemouth bass, *Micropterus salmoides*.

Substitution of 100% FM with PBM resulted in lipidosis with clearly visible inflammation in liver of juvenile tenches, *Tinca tinca* [39] which supported our present findings as hepatocyte lipid vacuolization with less amount of glycogen was observed in the liver tissue of barramundi fed PBM. The excessive amount of fat deposition in liver negatively impacted the growth and

immune response of fish [59] that are synchronous with immunological results in the present study. Similarly, Siddik, Chungu [32] fed juvenile barramundi with different levels of PBM over a period of 42 days and reported irregular liver arrangement with lipid deposition in the 100% PBM and bioprocessed PBM groups. Furthermore, higher administration of APB affected the morphology of liver of hybrid grouper, *Epinephelus fuscoguttatus*♀× *Epinephelus lanceolatus*♂, characterized by hepatic vacuoles and a high amounts of lipid droplets which is a sign of hepatic steatosis [18]. The lipid accumulation in liver may occur when dietary lipid exceeds the capacity of the hepatic cells to oxidize which lead to synthesize and deposit a larger amounts of triglyceride in vacuoles [18, 59, 60].

Muscle growth is the determinant of fish growth which can be affected by diet [61]. Nutritional deficiency could alter the muscle structure of Atlantic salmon, *Salmo salar* including myodigeneration [62]. Likewise, fish fed 100PBM showed necrosis and fibre degeneration in muscle which might be attributed by deficiency of essential PUFA. Gill is one of the important immune organs in fish and its structure can be affected by stress and diet [63]. In the present study, hyperplasia in secondary gill lamellae was in 100PBM fed fish but the possible reasons are not well understood which deserve further study.

Evaluating intestinal morphology in response to dietary changes is important to reflect the health status and welfare of fish. Intestinal morphology, in particular, villous structure, and microvillus height and diameter is related with absorption and assimilation of nutrient and immunological function [2, 64, 65]**.** In comparison with control, histological analysis showed that broken and short fold was found in the present study in 100PBM fed groups. In line with histological results, significantly smaller with shorter diameter of microvillus were observed, which might be responsible for lower efficiency of nutrient uptake and thus suppressing the growth and finally survival. Similar results were reported by Siddik, Howieson [2] who found significantly lower microvillus height in the distal intestine of barramundi after 56 days post-feeding with 10% supplemented 90PBM. Hence total replacement of FM with PBM impacted the welfare of juvenile barramundi, as reflected by the histology and TEM analysis.

Antioxidant status in fish, as determined by a number of antioxidants including CAT, SOD, GPx, and MDA have been considered as the first line of defensive biomarkers against oxidative damage. MDA is a natural biomarker and main ending product of lipid peroxidation [66, 67] and it elevation indicates oxidative injury [68] and associates with pathological state of animals including cell structure damage and function [66, 67]. Glutathione peroxidase, GPx, also an

important antioxidant enzymes play an important in preventing or repairing oxidative damage [69]. However, the concentration of MDA is well correlated with GPx activity [70], which are in agreement with the present findings. A lower concentration of MDA was found with a significant decrease in the serum of fish fed 100PBM, indicating that administration 100PBM had noticeable effect on the enzymatic antioxidant activity of fish. The mechanisms behind negative effects is not well understood which deserve further study. However, inclusion of 60% cottonseed meal depressed the antioxidant activity (SOD, CAT, GSH-Px, TAC and MDA) in in liver of Ussuri catfish, *Pseudobagrus ussuriensis* at the end of 56 days feeding trial [71].

In summary, regardless of methionine supplementation, the total replacement of FM with PBM is not nutritionally adequate for juvenile barramundi, as indicated by depressed growth performance and immune response, as well as elevated level of MDA activity. Also, adding PBM induced the lipid droplet in liver for juvenile barramundi via modulating the expression levels of heat shock related genes and liver enzymes. Feeding PBM not only triggered the fibre degeneration and necrosis in muscle and hyperplasia in gills, but also induced the intestinal villus morphology by decreasing intestinal microvilli morphology, which may suggest that high levels of PBM could impair the welfare of juvenile barramundi. Further long term study need to be conducted along with supplementation of other EAA and/or EFA with PBM to investigate the welfare of farmed juvenile barramundi.

Acknowledgements

Support of the trial was obtained from the Research Training Program (RTP) Stipend Scholarship, funded by Australian Government to Md Reaz Chaklader (No. 19061054-Curtin). The authors are also thankful to Australian Centre for Applied Aquaculture Research (ACAAR), Fremantle, Australia for providing fish and Rowan Kleindienst for technical assistance during fish husbandry. We are also sincerely thankful to Dr Stephens Fran for helping to collect histological samples and prepare histological slides.

Data availability: All datasets generated during the present study have been presented in the form of figures and tables but are available from the corresponding author on reasonable request.

Competing Interests: The authors declare no competing interests.

References

1. Bharadwaj AS, Brignon WR, Gould NL, Brown PB, Wu YV. Evaluation of Meat and Bone Meal in Practical Diets Fed to Juvenile Hybrid Striped Bass Morone chrysops × M. saxatilis. Journal of the World Aquaculture Society. 2002;33(4):448-57. doi: 10.1111/j.1749-7345.2002.tb00024.x.

2. Siddik MAB, Howieson J, Fotedar R. Beneficial effects of tuna hydrolysate in poultry by-product meal diets on growth, immune response, intestinal health and disease resistance to *Vibrio harveyi* in juvenile barramundi, *Lates calcarifer*. Fish and Shellfish Immunology. 2019. doi: https://doi.org/10.1016/j.fsi.2019.03.042.

3. Kang Y, Lee S, Yang S, Bai S. Effects of meat meal, blood meal or soybean meal as a dietary protein source replacingfish meal in parrotfish,*Oplegnathus fasciatus*. Journal of Aquaculture. 1999;12:205–12.

4. González- Rodríguez Á, Celada JD, Carral JM, Sáez- Royuela M, García V, Fuertes JB. Evaluation of poultry by- product meal as partial replacement of fish meal in practical diets for juvenile tench (Tinca tinca L. Aquaculture Research. 2016;47(5):1612-21. doi: 10.1111/are.12622.

5. Zhou Q-C, Zhao J, Li P, Wang H-L, Wang L-G. Evaluation of poultry by-product meal in commercial diets for juvenile cobia (*Rachycentron canadum*). Aquaculture. 2011;322-323:122-7. doi: 10.1016/j.aquaculture.2011.09.042.

6. Zapata DB, Lazo JP, Herzka SZ, Viana MT. The effect of substituting fishmeal with poultry by- product meal in diets for Totoaba macdonaldi juveniles. Aquaculture Research. 2016;47(6):1778-89. doi: 10.1111/are.12636.

7. Parés- Sierra G, Durazo E, Ponce MA, Badillo D, Correa- Reyes G, Viana MT. Partial to total replacement of fishmeal by poultry by- product meal in diets for juvenile rainbow trout (Oncorhynchus mykiss) and their effect on fatty acids from muscle tissue and the time required to retrieve the effect. Aquaculture Research. 2014;45(9):1459-69. doi: 10.1111/are.12092.

8. Turker A, Yigit M, Ergun S, Karaali B, Erteken A. Potential of poultry byproduct meal as a substitute forfishmeal in diets for Black Sea turbot *Scophthalmus maeoticus*: growth and nutrient utilization in winter. Israeli Journal of AquacultureBamidgeh. 2005;57:49–61.

9. Wang Y, Guo JL, Bureau DP, Cui ZH. Replacement of fish meal by rendered animal protein ingredients in feeds for cuneate drum (Nibea miichthioides). Aquaculture. 2006;252:476-83.

10. Gaylord TG, Rawles SD. The Modification of Poultry By- product Meal for Use in Hybrid Striped Bass Morone chrysops × M. saxatilis Diets. Journal of the World Aquaculture Society. 2005;36(3):363-74. doi: 10.1111/j.1749-7345.2005.tb00340.x.

11. Yigit M, Erdem M, Koshio S, Ergün S, Türker A, Karaali B. Substituting fish meal with poultry by- product meal in diets for black Sea turbot *Psetta maeotica*. Aquaculture Nutrition. 2006;12(5):340-7. doi: 10.1111/j.1365-2095.2006.00409.x.

12. Zhou Q-C, Zhao J, Li P, Wang H-L, Wang L-G. Evaluation of poultry by-product meal in commercial diets for juvenile cobia (Rachycentron canadum). Aquaculture. 2011;322:122-7. doi: 10.1016/j.aquaculture.2011.09.042.

13. Karapanagiotidis IT, Psofakis P, Mente E, Malandrakis E, Golomazou E. Effect of fishmeal replacement by poultry by-product meal on growth performance, proximate composition, digestive enzyme activity, haematological parameters and gene expression of gilthead seabream (Sparus aurata). Aquaculture Nutrition. 2019;25(1):3-14. doi: 10.1111/anu.12824.

14. Shapawi R, Ng W-K, Mustafa S. Replacement of fish meal with poultry by-product meal in diets formulated for the humpback grouper, *Cromileptes altivelis*. Aquaculture. 2007;273(1):118-26. doi: 10.1016/j.aquaculture.2007.09.014.

15. Michelato M, Furuya WM, Gatlin DM. Metabolic responses of Nile tilapia Oreochromis niloticus to methionine and taurine supplementation. Aquaculture. 2018;485:66-72. doi: 10.1016/j.aquaculture.2017.11.003.

16. Espe M, Liaset B, Hevroey E, El-Mowafi A. dl-methionine enrichment in diets fed to Atlantic salmon increases apparent digestibility. Aquaculture Research. 2011;42(8):1123-30. doi: 10.1111/j.1365-2109.2010.02700.x.

17. de Moura LB, Diógenes AF, Campelo DAV, Almeida FLAd, Pousão-Ferreira PM, Furuya WM, et al. Taurine and methionine supplementation as a nutritional strategy for growth promotion of meagre (Argyrosomus regius) fed high plant protein diets. Aquaculture. 2018;497:389-95. doi: 10.1016/j.aquaculture.2018.07.038.

18. Ye H, Zhou Y, Su N, Wang A, Tan X, Sun Z, et al. Effects of replacing fish meal with rendered animal protein blend on growth performance, hepatic steatosis and immune status in hybrid grouper (Epinephelus fuscoguttatus♀ × Epinephelus lanceolatus♂). Aquaculture. 2019;511:734203. doi: 10.1016/j.aquaculture.2019.734203.

19. Campos I, Matos E, Marques A, Valente LMP. Hydrolyzed feather meal as a partial fishmeal replacement in diets for European seabass (Dicentrarchus labrax) juveniles. Aquaculture. 2017;476:152-9. doi: 10.1016/j.aquaculture.2017.04.024.

20. Wang L, Zhou H, He R, Xu W, Mai K, He G. Effects of soybean meal fermentation by Lactobacillus plantarum P8 on growth, immune responses, and intestinal morphology in juvenile turbot (Scophthalmus maximus L.). Aquaculture. 2016;464:87-94. doi: 10.1016/j.aquaculture.2016.06.026.

21. Khosravi S, Rahimnejad S, Herault M, Fournier V, Lee C-R, Dio Bui HT, et al. Effects of protein hydrolysates supplementation in low fish meal diets on growth performance, innate immunity and disease resistance of red sea bream Pagrus major. Fish and Shellfish Immunology. 2015;45(2):858-68. doi: 10.1016/j.fsi.2015.05.039.

22. Kokou F, Rigos G, Henry M, Kentouri M, Alexis M. Growth performance, feed utilization and non-specific immune response of gilthead sea bream (Sparus aurata L.) fed graded levels of a bioprocessed soybean meal. Aquaculture. 2012;364-365:74-81. doi: 10.1016/j.aquaculture.2012.08.009.

23. Rawles S, Thompson K, Brady Y, Metts L, Aksoy M, Gannam AL, et al. Effects of replacing fish meal with poultry byaproduct meal and soybean meal and reduced protein level on the performance and immune status of pondagrown sunshine bass (*Morone chrysops M. saxatilis*). Aquaculture Nutrition. 2011;17(3):708-21. doi: 10.1111/j.1365-2095.2010.00831.x.

24. Palupi ET, Setiawati M, Lumlertdacha S, Suprayudi MA. Growth performance, digestibility, and blood biochemical parameters of Nile tilapia (Oreochromis niloticus) reared in floating cages and fed poultry by-product meal. Journal of Applied Aquaculture. 2019:1-18. doi: 10.1080/10454438.2019.1605324.

25. Zhu H, Gong G, Wang J, Wu X, Xue M, Niu C, et al. Replacement of fish meal with blend of rendered animal protein in diets for Siberian sturgeon (Acipenser baerii Brandt), results in performance equal to fish meal fed fish. Aquaculture nutrition. 2011;17(2):e389-e95. doi: 10.1111/j.1365-2095.2010.00773.x.

26. Huntingford FA, Kadri S. Defining, assessing and promoting the welfare of farmed fish. . Revue Scientifique et Technique (International Office of Epizootics). 2014;33:pp. 233–44.

27. Saraiva A, Costa J, Serrão J, Cruz C, Eiras J. A histology-based fish health assessment of farmed seabass (Dicentrarchus labrax L.). Aquaculture. 2015;448:375.

28. Tort L, Pavlidis MA, Woo NYS. Stress and welfare in sparidfishes. In: Pavlidis, M.A., Mylonas, C.C. (Eds.), Sparidae Biology and Aquaculture of Gilthead Sea Bream and Other Species. Wiley-Blackwell, Chichester. 2011:pp. 75–94.

29. Lukin A, Sharova J, Belicheva L, Camus L. Assessment of fish health status in the Pechora River: effects of contamination. Ecotoxicology and environmental safety. 2011;74(3):355. doi: 10.1016/j.ecoenv.2010.10.022.

30. McHugh KJ, Smit NJ, Van Vuren JHJ, Van Dyk JC, Bervoets L, Covaci A, et al. A histology-based fish health assessment of the tigerfish, Hydrocynus vittatus from a DDT-affected area. Physics and Chemistry of the Earth. 2011;36(14-15):895-904. doi: 10.1016/j.pce.2011.07.077.

31. McHugh KJ, Dyk JC, Weyl OLF, Smit NJ. First report of nephrocalcinosis in a wild population of M ugil cephalus L. and M yxus capensis (V alenciennes. Journal of Fish Diseases. 2013;36(10):887-9. doi: 10.1111/jfd.12101.

32. Siddik M, Chungu P, Fotedar R, Howieson J. Bioprocessed poultry by-product meals on growth, gut health and fatty acid synthesis of juvenile barramundi, *Lates calcarifer* (Bloch). PLoS One. 2019;14(4):e0215025. doi: 10.1371/journal.pone.0215025. PubMed PMID: TN_proquest2208024522.

33. Simon CJ, Salini MJ, Irvin S, Blyth D, Bourne N, Smullen R. The effect of poultry protein concentrate and phosphorus supplementation on growth, digestibility and nutrient retention efficiency in barramundi Lates calcarifer. Aquaculture. 2019;498:305-14. doi: 10.1016/j.aquaculture.2018.08.069.

34. NRC. Nutrient Requirements of Fish and Shrimp: National Academies Press; 2011.

35. Siddik MAB, Howieson J, Partridge GJ, Fotedar R, Gholipourkanani H. Dietary tuna hydrolysate modulates growth performance, immune response, intestinal morphology and resistance to Streptococcus iniae in juvenile barramundi, *Lates calcarifer*. Sci Rep. 2018;8(1):15942. Epub 2018/10/31. doi: 10.1038/s41598-018-34182-4. PubMed PMID: 30374125; PubMed Central PMCID: PMCPMC6206086.

36. O'Fallon JV, Busboom JR, Nelson ML, Gaskins CT. direct method for fatty acid methyl ester synthesis: Application to wet meat tissues, oils, and feedstuffs. direct method for fatty acid methyl ester synthesis: Application to wet meat tissues, oils, and feedstuffs. 2007;85(6):1511-21. doi: 10.2527/jas.2006-491.

37. Chaklader MR, Siddik MAB, Fotedar R, Howieson J. Insect larvae, Hermetia illucens in poultry by-product meal for barramundi, *Lates calcarifer* modulates histomorphology, immunity and resistance to *Vibrio harveyi*. Scientific reports. 2019;9:16703.

38. Mohd-Shaharuddin N, Mohd-Adnan A, Kua B-C, Nathan S. Expression profile of immune-related genes in *Lates calcarifer* infected by Cryptocaryon irritans. Fish & shellfish immunology. 2013;34(3):762-9. doi: 10.1016/j.fsi.2012.11.052.

39. Panicz R, Żochowska- Kujawska J, Sadowski J, Sobczak M. Effect of feeding various levels of poultry by- product meal on the blood parameters, filet composition and structure of female tenches (Tinca tinca. Aquaculture Research. 2017;48(10):5373-84. doi: 10.1111/are.13351.

40. Hu M, Wang Y, Wang Q, Zhao M, Xiong B, Qian X, et al. Replacement of fish meal by rendered animal protein ingredients with lysine and methionine supplementation to practical diets for gibel carp, Carassius auratus gibelio. Aquaculture. 2008;275(1-4):260-5. doi: 10.1016/j.aquaculture.2008.01.005.

41. Higgs DA, Balfry SK, Oakes JD, Rowshandeli M, Skura BJ, Deacon G. Efficacy of an equal blend of canola oil and poultry fat as an alternate dietary lipid source for Atlantic salmon (Salmo salar L.) in sea water. I: effects on growth performance, and whole body and fillet proximate and lipid composition. Aquaculture Research. 2006;37(2):180-91. doi: 10.1111/j.1365-2109.2005.01420.x.

42. Turchini GM, Torstensen BE, Ng WK. Fish oil replacement in finfish nutrition. Reviews in Aquaculture. 2009;1(1):10-57. doi: 10.1111/j.1753-5131.2008.01001.x.

43. Emre Y, Kurtoğlu A, Emre N, Güroy B, Güroy D. Effect of replacing dietary fish oil with soybean oil on growth performance, fatty acid composition and haematological parameters of juvenile meagre, Argyrosomus regius. Aquaculture Research. 2016;47(7):2256-65. doi: 10.1111/are.12677.

44. González-Félix ML, Maldonado-Othón CA, Perez-Velazquez M. Effect of dietary lipid level and replacement of fish oil by soybean oil in compound feeds for the shortfin corvina (Cynoscion parvipinnis). Aquaculture. 2016;454:217-28. doi: 10.1016/j.aquaculture.2015.12.021.

45. Xu H, Zhang Y, Wang J, Zuo R, Mai K, Ai Q. Replacement of Fish Oil with Linseed Oil or Soybean Oil in Feeds for Japanese Seabass, Lateolabrax japonicus: Effects on Growth Performance, Immune Response, and Tissue Fatty Acid Composition. Journal of the World Aquaculture Society. 2015;46(4):349-62. doi: 10.1111/jwas.12205.

46. Jacobsen C. Enrichment of foods with omega- 3 fatty acids: a multidisciplinary challenge. Annals of the New York Academy of Sciences. 2010;1190(1):141-50. doi: 10.1111/j.1749-6632.2009.05263.x.

47. Mensink R. Effects of Saturated Fatty Acids on Serum Lipids and Lipoproteins: A Systematic Review and Regression Analysis. World Health Organization, Geneva. 2016.

48. Michielsen CCJR, Hangelbroek RWJ, Feskens EJM, Afman LA. Disentangling the Effects of Monounsaturated Fatty Acids from Other Components of a Mediterranean Diet on Serum Metabolite Profiles: A Randomized Fully Controlled Dietary Intervention in Healthy Subjects at Risk of the Metabolic Syndrome. Molecular Nutrition & Food Research. 2019;63(9):n/a-n/a. doi: 10.1002/mnfr.201801095.

49. Blondeau N, Lipsky RH, Bourourou M, Duncan MW, Gorelick PB, Marini AM. Alpha-Linolenic Acid: An Omega-3 Fatty Acid with Neuroprotective Properties—Ready for Use in the Stroke Clinic? BioMed Research International. 2015;2015. doi: 10.1155/2015/519830.

50. Renna M, Schiavone A, Gai F, Dabbou S, Lussiana C, Malfatto V, et al. Evaluation of the suitability of a partially defatted black soldier fly (L.) larvae meal as ingredient for rainbow trout (Walbaum) diets. Journal of animal science and biotechnology. 2017;8(1):57. doi: 10.1186/s40104-017-0191-3.

51. Huang L, Ran C, He S, Ren P, Hu J, Zhao X, et al. Effects of dietary Saccharomyces cerevisiae culture or live cells with Bacillus amyloliquefaciens spores on growth performance, gut mucosal morphology, hsp70 gene expression, and disease resistance of juvenile common carp (Cyprinus carpio). Aquaculture. 2015;438:33-8. doi: 10.1016/j.aquaculture.2014.12.029.

52. Rollo A, Sulpizio R, Nardi M, Silvi S, Orpianesi C, Caggiano M, et al. Live microbial feed supplement in aquaculture for improvement of stress tolerance. Fish Physiology and Biochemistry. 2006;32(2):167-77. doi: 10.1007/s10695-006-0009-2.

53. Ming J, Xie J, Xu P, Ge X, Liu W, Ye J. Effects of emodin and vitamin C on growth performance, biochemical parameters and two HSP70s mRNA expression of Wuchang bream (Megalobrama amblycephala Yih) under high temperature stress. Fish and Shellfish Immunology. 2012;32(5):651-61. doi: 10.1016/j.fsi.2012.01.008.

54. Burgos-Aceves MA, Cohen A, Smith Y, Faggio C. Estrogen regulation of gene expression in the teleost fish immune system. Fish and Shellfish Immunology. 2016;58:42-9. doi: 10.1016/j.fsi.2016.09.006.

55. Lauriano ER, Pergolizzi S, Capillo G, Kuciel M, Alesci A, Faggio C. Immunohistochemical characterization of Toll-like receptor 2 in gut epithelial cells and macrophages of goldfish Carassius auratus fed with a high-cholesterol diet. Fish and Shellfish Immunology. 2016;59(C):250-5. doi: 10.1016/j.fsi.2016.11.003.

56. Lazado CC, Caipang CMA. Mucosal immunity and probiotics in fish. Fish and Shellfish Immunology. 2014;39(1):78-89. doi: 10.1016/j.fsi.2014.04.015.

57. Subhadra B, Lochmann R, Rawles S, Chen R. Effect of dietary lipid source on the growth, tissue composition and hematological parameters of largemouth bass (Micropterus salmoides). Aquaculture. 2006;255:210-22.

58. Subhadra B, Lochmann R, Rawles S, Chen R. Effect of fish-meal replacement with poultry by-product meal on the growth, tissue composition and hematological parameters of largemouth bass (Micropterus salmoides) fed diets containing different lipids. Aquaculture. 2006;260(1-4):221-31. doi: 10.1016/j.aquaculture.2006.06.029.

59. Kang-Le L, Wei-Na X, Li-Na W, Ding-Dong Z, Chun-Nuan Z, Wen-Bin L. Hepatic β-oxidation and regulation of carnitine palmitoyltransferase (CPT) I in blunt snout bream Megalobrama amblycephala fed a high fat diet. PLoS ONE. 2014;9(3):e93135. doi: 10.1371/journal.pone.0093135.

60. Spisni E, Tugnoli M, Ponticelli A, Mordenti T, Tomasi V. Hepatic steatosis in artificially fed marine teleosts. Journal of Fish Diseases. 1998;21(3):177-84. doi: 10.1046/j.1365-2761.1998.00089.x.

61. Alami-Durante H, Bazin D, Cluzeaud M, Fontagné-Dicharry S, Kaushik S, Geurden I. Effect of dietary methionine level on muscle growth mechanisms in juvenile rainbow trout (Oncorhynchus mykiss). Aquaculture. 2018;483:273-85. doi: 10.1016/j.aquaculture.2017.10.030.

62. Rodger H, Murphy T, Drinan E, Rice D. Acute skeletal myopathy in farmed Atlantic salmon Salmo salar. Diseases of Aquatic Organisms. 1991;12(1):17-23.

63. Anderson Brunetti R, Débora de Mello Gonçales SA, Jorge Fernandes de A, Luiz Sérgio M, Eduardo José de Almeida A. Alterações do epitélio branquial e das lamelas de tilápias (Oreochromis niloticus) causadas por mudanças do ambiente aquático em tanques de cultivo intensivo The influence of the aquatic environment in tanks sequetially interconnected with PVC pipes on the gill epithelium and lamellas of tilapia (Oreochromis niloticus). Pesquisa Veterinária Brasileira. 2009;29(4):303-11. doi: 10.1590/S0100-736X2009000400005.

64. Nicholson JK, Holmes E, Kinross J, Burcelin R, Gibson G, Jia W, et al. Host-gut microbiota metabolic interactions. Science (New York, NY). 2012;336(6086):1262. doi: 10.1126/science.1223813.

65. Urán PA, Schrama JW, Rombout JHWM, Obach A, Jensen L, Koppe W, et al. Soybean meal- induced enteritis in Atlantic salmon (Salmo salar L.) at different temperatures. Aquaculture Nutrition. 2008;14(4):324-30. doi: 10.1111/j.1365-2095.2007.00534.x.

66. Nugroho R, Fotedar R. Comparing the effects of dietary selenium and mannan oligosaccharide supplementation on the growth, immune function, and antioxidant enzyme activity in the cultured marron Cherax cainii (Austin, 2002). Journal of the European Aquaculture Society. 2014;22(2):585-96. doi: 10.1007/s10499-013-9682-1.

67. Satoshi S, Kiyoji T, Hiroyo K, Fumio N. Exercise-induced lipid peroxidation and leakage of enzymes before and after vitamin E supplementation. International Journal of Biochemistry. 1989;21(8):835-8. doi: 10.1016/0020-711X(89)90280-2.

68. Halliwell B, Gutteridge JMC. Free Radicals in Biology and Medicine,fifth ed. Oxford University Press, Croydon, UK. 2015:pp. 944.

69. Wang W-N, Zhou J, Wang P, Tian T-T, Zheng Y, Liu Y, et al. Oxidative stress, DNA damage and antioxidant enzyme gene expression in the Pacific white shrimp, Litopenaeus vannamei when exposed to acute pH stress. Comparative Biochemistry and Physiology, Part C. 2009;150(4):428-35. doi: 10.1016/j.cbpc.2009.06.010.

70. Sabzi E, Mohammadiazarm H, Salati AP. Effect of dietary l-carnitine and lipid levels on growth performance, blood biochemical parameters and antioxidant status in juvenile common carp (Cyprinus carpio). Aquaculture. 2017;480:89-93. doi: 10.1016/j.aquaculture.2017.08.013.

71. Bu X, Chen A, Lian X, Chen F, Zhang Y, Muhammad I, et al. An evaluation of replacing fish meal with cottonseed meal in the diet of juvenile Ussuri catfish Pseudobagrus ussuriensis: Growth, antioxidant capacity, nonspecific immunity and resistance to Aeromonas hydrophila. Aquaculture. 2017;479:829-37. doi: 10.1016/j.aquaculture.2017.07.032.
