# Supplementary material for: Total replacement of fishmeal with poultry by-product meal affected the growth, muscle quality, histological structure, antioxidant capacity and immune response of juvenile barramundi, Lates calcarifer
Source: PLoS One. 2020 Nov 12;15(11):e0242079. doi: 10.1371/journal.pone.0242079 (PMC7661056; doi:10.1371/journal.pone.0242079)
Supplement: Attachment — Submitted filename: Plos One Review PONE.pdf [file pone.0242079.s002.pdf]

Plos One Review PONE-D-20-07405

Total replacement of fishmeal with poultry by-product meal affected the growth, muscle quality, histological structure, antioxidant capacity and immune response of juvenile barramundi, Lates calcarifer

Authors: Md Reaz Chaklader[1*], Muhammad A.B. Siddik[2], Ravi Fotedar[1]

General comments:

The authors present an interesting and well-constructed manuscript evaluating fishmeal and poultry meal based diets fed to juvenile barramundi. There are consistent issues throughout with the writing style in a scientific contest, the description of results, discussion and conclusions need to be improved. Quiet clear is the lack of appropriate citation and relevance to the study.

My most obvious concern with the work is the lack of understanding in the field, in particular with the species in question. The barramundi is a very well understood species, with a lot of very old publication and work undertaken by various labs predominantly in Australian and south east Asia. This dates back around 30 years. I can find specific reference to the use of poultry meal in barramundi diets published in 1998. So, this is by no means a novel raw material for barramundi. The use of terrestrial meals as protein in barramundi is fundamentally well understood, and taking a deep dive we can see that there are studies that even evaluate multiple poultry meal types and their level of processing on the performance of barramundi. So this is not a new area of research. Moreover, their selection of relevant references is odd given there are numerous papers specifically on barramundi yet they chose to cite authors working on vastly different fish in the introduction. Its just a comment rather than criticism but I think should be revised.

46 *M. Boonyaratpalin and K. Williams*

Table 3.2. Apparent protein and energy digestibility coefficients of major protein feed ingredients in sea-bass diets (from Williams *et al.*, 1998).

	Digestibility coefficients (%)*	
Feed ingredient	Protein	Energy
Danish fish-meal	87.9 ± 1.0	83.3 ± 1.3
Tuna fish-meal	92.3 ± 1.0	69.3 ± 1.3
Poultry offal meal	78.8 ± 3.5	76.7 ± 5.6
Meat meal (high ash)	53.9 ± 3.9	58.2 ± 6.5
Meat meal (low ash)	63.5 ± 3.4	66.5 ± 3.4
Soybean meal (full-fat)	84.8 ± 3.8	75.9 ± 7.8
Soybean meal (solvent-extracted)	86.0 ± 0.8	69.4 ± 1.7
Canola meal	81.0 ± 2.3	56.1 ± 3.0
Lupin meal (dehulled)	98.1 ± 1.3	61.5 ± 1.8
Groundnut meal	91.9 ± 8.0	68.7 ± 5.0
Wheat-gluten meal	101.9 ± 1.6	98.8 ± 3.1

* Mean ± standard error. Data derived from faeces collected either by hand-stripping or by intestinal dissection.

Introduction

The introduction is written in a way that requires attention. It is going all over the place from rendered animal meals, unusual reference to unrelated obscure species, LC-PUFAs, lysine content,

growth hormones, methionine metabolism, health status (immune, antioxidant), aquatic pollution. This distracts from the main purpose and history of R&D on barramundi in Australia and elsewhere.

Methods

- The Diet formulas are presented in table 1, however the title indicates the test diets were supplemented with protein hydrolysates. This is clearly from another study and cut and paste.
- The Fatty Acid analysis section lacks a lot of detail.
- Ive never seen fatty acids and amino acids presented side by side like that. Its odd.
- Antioxidant status assessment lacks a lot of detail
- In the calculation, there are 2 things requiring attention, AI and TI.

Results

- You really need a table of results here that clearly show the values +/- SE for your data. The figure 1 is not satisfactory. FBG acronym not explained.
- Fig 1 appears to have individual fish (n=64) in some and tank replication in the other (n=3). Not clear as to why this is done differently.
- Muscle FA section poorly written.
- Table 3. Units? Also, check stats. Row C18:1cis+trans are very different values whereas C17:0 are almost the same (44 v 40) yet highly significant.
- Table 3. What have AI and TI got to do with the FAME?
- In Histomorphology no mention of the gill and intestine result (Fig 2)

Discussion

- A few studies? There are dozens specifically investigating this material in fish and prawns.
- Have they already done this study ref 32?
- In terms of Methionine supplementation, the authors ended up with different levels of Met in the diets despite their attempts to balance. Either way the inclusion in both diets is well above the known requirement for the species and I doubt this would be a relevant reason for the differences observed. Need to discuss the results in more detail with a focus on the things that are explainable.
- Similarly, with EFA story there are reports on fatty acids nutrition specifically in barramundi but these have largely been ignored in favour of other more obscure species. Moreover, this study is primarily an evaluation of a protein rich ingredient to replace fishmeal, so we are crossing over a lot here with lipid specific studies.
- Generally, the authors are describing their methods here but not relating it to or explaining in light of the present study. Take the CAT, SOD, GPX section for example.

Acknowlegements

- Its Dr Fran Stephens. This clearly indicates your lack of understanding and probably had a lot of help. Fran is an expert in her field and should at least have her name correctly acknowledged. If she has helped in this analysis and interpretation of data, maybe consider her as a co-author?
