# Supplementary material for: Total replacement of fishmeal with poultry by-product meal affected the growth, muscle quality, histological structure, antioxidant capacity and immune response of juvenile barramundi, Lates calcarifer
Source: PLoS One. 2020 Nov 12;15(11):e0242079. doi: 10.1371/journal.pone.0242079 (PMC7661056; doi:10.1371/journal.pone.0242079)
Supplement: Attachment — Submitted filename: Response to Reviewers.docx [file pone.0242079.s004.docx]

**Response to Reviewer 1**

Reviewer #1: This revision is greatly improved. Well done. If i had to be picky i would have said that the methionine requirement in barramundi was revised a few years back and there are a couple of references on the subject, so the older reference #8 cited is actually quite outdated. Having said that the MS reads well and is ready for publication.

Response: We have added updated reference. Please see line 151.

**Response to Reviewer 3**

Reviewer #3: The Manuscript is well designed and conducted and presents an interesting subject to the marine aquaculture industry. However, it did not tell the optimum inclusion level of poultry by product meal with and without methionine supplementation, which is a very important issue for the journal readers and for application.

Minor comments
The proximate composition of diets lacks information on the mineral contents and energy values. Also, all values in Tables 1-3 miss +/- SE (SD)

**Response:**

Thank you for your complements regarding the design of the experiment and its conduction. Our published earlier article (Chaklader et al., 2020) has proved that 90% poultry by product meal along with the supplementation of fish protein hydrolysate can replace the fishmeal with some positive physiological outcomes for barramundi. As we found that methionine was lacking in the poultry by product meal for enhancing the growth of barramundi and therefore the current study was aimed to investigate if methionine supplementation alone in poultry by product meal could replace fishmeal entirely from the diet of barramundi. However, the current results clearly indicated that methionine alone is not able to replace the contribution of fish protein hydrolysate in barramundi diet. As fish protein hydrolysate have other nutrient ingredients (such as biologically active low molecular weight peptides) those in combination with poultry by product meal are able to replace fishmeal entirely in barramundi diets.

The results of previously published article and current article under review clearly indicate although methionine is essential for barramundi’s physiological performance, fish hydrolysate in addition to methionine has other beneficial micro nutrient embedded in it. Hence, optimum level of poultry by product meal in barramundi diet is 90% of the protein source along with fish hydrolysate without any addition of pure methionine. The optimum inclusion level of poultry by product meal with purified methionine supplementation is yet to be investigated.

Chaklader MR, Fotedar R, Howieson J, Siddik MAB, Foysal MJ. 2020. The ameliorative effects of various fish protein hydrolysates in poultry by-product meal diets on muscle quality, serum biochemistry and immunity in juvenile barramundi*, Lates calcarifer. Fish & Shellfish Immunology*, *104:*567-578. <https://doi.org/10.1016/j.fsi.2020.06.014>

Proximate composition has been revised (Table 1). Table 1 is the calculation of feed formulation with analysed proximate composition while amino acid (Table 2) and fatty acid composition (Table 3) was analysed for one sample repeated in triplicate. One sample/treatment particularly for diets is accepted in aquaculture study as it is depicted in various publication (Siddik et al., 2019, Chaklader 2020, Ye et al., 2019 and Sabbagh et al., 2020).

Siddik MAB, Chungu P, Fotedar R, Howieson J (2019) Bioprocessed poultry byproduct meals on growth, gut health and fatty acid synthesis of juvenile barramundi, *Lates calcarifer* (Bloch). PLoS ONE 14(4): e0215025. https://doi. org/10.1371/journal.pone.0215025

Chaklader MR, Fotedar R, Howieson J, Siddik MAB, Foysal MJ. 2020. The ameliorative effects of various fish protein hydrolysates in poultry by-product meal diets on muscle quality, serum biochemistry and immunity in juvenile barramundi*, Lates calcarifer. Fish & Shellfish Immunology*, *104:*567-578. <https://doi.org/10.1016/j.fsi.2020.06.014>

Ye, H., Zhou, Y., Su, N., Wang, A., Tan, X., Sun, Z., Zou, C., Liu, Q., Ye, C., 2019. Effects of replacing fish meal with rendered animal protein blend on growth performance, hepatic steatosis and immune status in hybrid grouper (Epinephelus fuscoguttatus♀ × Epinephelus lanceolatus♂). Aquaculture 511, 734203. 10.1016/j.aquaculture.2019.734203.

Sabbagh, M., Schiavone, R., Brizzi, G., Sicuro, B., Zilli, L., Vilella, S., 2019. Poultry by-product meal as an alternative to fish meal in the juvenile gilthead seabream (Sparus aurata) diet. Aquaculture 511, 734220.
